# Clinical Application of Forced Oscillation Technique (FOT) in Early Detection of Airway Changes in Smokers

**DOI:** 10.3390/jcm9092778

**Published:** 2020-08-27

**Authors:** Prem Bhattarai, Stephen Myers, Collin Chia, Heinrich C. Weber, Sally Young, Andrew D. Williams, Sukhwinder Singh Sohal

**Affiliations:** 1 Respiratory Translational Research Group, Department of Laboratory Medicine, School of Health Sciences, University of Tasmania, Launceston, Tasmania 7248, Australia; Prem.Bhattarai@utas.edu.au (P.B.); Stephen.Myers@utas.edu.au (S.M.); collin.chia@ths.tas.gov.au (C.C.); heinrich.weber@ths.tas.gov.au (H.C.W.); sally.young@ths.tas.gov.au (S.Y.); andrew.williams@utas.edu.au (A.D.W.); 2 Department of Respiratory Medicine, Launceston General Hospital, Launceston, Tasmania 7250, Australia; 3 Department of Respiratory Medicine, Tasmanian Health Services (THS), North West Hospital, Burnie, Tasmania 7320, Australia; 4 Lung Function Unit, North West Regional Hospital, Burnie, Tasmania 7320, Australia

**Keywords:** COPD, forced oscillation technique, smoking, small airways

## Abstract

The forced oscillation technique (FOT) is a non-invasive method to assess airway function by emitting oscillatory signals into the respiratory tract during tidal ventilation. This opinion piece discusses the current use, trialled modification and future directions in utilizing FOT as a novel diagnostic tool for early detection of small airway changes in smokers. The published evidence to date has shown that FOT parameters could be a sensitive diagnostic tool to detect early respiratory changes in smokers. Multiple frequencies and the frequency dependence of resistance and reactance can provide the most valuable and early information regarding smoking induced changes in airways. Considering its non-invasiveness, lower level of discomfort to patients than spirometry, feasibility, and cost effectiveness, it could be the first-choice diagnostic technique for detection of early respiratory changes in smokers. The finding of FOT could further be supported and correlated with inflammatory markers.

## 1. Background

Tobacco smoke exposure is the second leading risk factor for early death and disability and has been directly responsible for more than 5 million deaths every year since 1990 [1,2]. The deterioration in pulmonary function seen in chronic obstructive pulmonary disease (COPD) is related to smoke inhalation by the patient comprising duration and number of smoking pack years as well as exposure to other sources of smoke pollution such as biomass fuel [3,4,5,6]. Dysfunction of the peripheral airways, lung parenchyma and microvasculature are common in smokers. [7,8]. It is believed that small airways (<2 mm) are the initial sites of inflammation and destruction in COPD and represent the silent zone of lung disease with earlier damage occurring without leading to observable airflow obstruction or symptoms [9,10,11]. The clinical consequences of these changes remained unexplored, potentially due to the lack of sensitive diagnostic tests to measure small airway function and the capacity of the human respiratory system to apparently function normally until the majority of airways become abnormal [8].

In routine clinical practice the alterations in respiratory function, due to smoking or other noxious stimuli, are evaluated using spirometry. However, spirometry is not sensitive enough to detect early lung function abnormalities [12,13,14]. Moreover, spirometry requires a number of very rigorous and effort-dependent breathing manoeuvres to obtain reliable results which can make it challenging for elderly patients and those with motor or cognitive impairment [12]. It is now extensively believed that newer technologies that can detect the early structural and functional changes in small airways before its progression to symptomatic COPD would contribute in decreasing medical and economic burdens by allowing us to develop the appropriate strategies for prevention or abrogation of the long-term sequelae and health care consequences of COPD [15,16,17].

**Figure 1:**
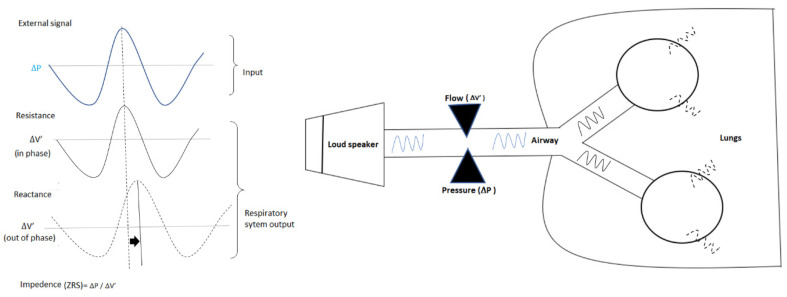
Schematic representation of input signal and output response by respiratory system in forced oscillation technique (FOT).

The forced oscillometry technique (FOT, Figure 1) first described by Dubios et al. in 1956 is a non-invasive approach to investigate mechanical properties of the respiratory system by assessing airway impedance (pressure/flow signal) after emitting oscillatory pressures of different frequencies into the respiratory tract during tidal ventilation [18]. Studies with modern FOT devices have indicated that FOT could be a more sensitive and versatile diagnostic tool to detect pre-COPD and COPD changes including expiratory flow limitation and functional inhomogeneity [19,20,21,22,23]. FOT has also shown the potential to sensitively detect precocious damage to respiratory system in young passive smokers compared to non-smokers [24]. There are several other articles on the clinical application of FOT analysis in respiratory practice but the majority of these studies are focused on large airway function and diagnosis of asthma [14,25,26,27,28,29,30]. However, the effectiveness of this technique in identifying early abnormalities in small airways has not been described in detail and there is still paucity of data regarding the clinical usefulness of FOT indices for early detection of smoking induced respiratory changes.

Although there are a few original articles evaluating the usefulness of FOT in detecting early smoking-related changes in lungs, it is extremely difficult to draw any conclusions from these studies (Summary Table 1). There is no agreement on the findings of these studies regarding the sensitivity and reliability of different parameters to detect small airway abnormalities in smokers, further the studies did not use comparable oscillation techniques [19,20,31,32,33,34,35,36,37,38,39]. Accordingly, it is timely to highlight these discrepancies and review the current status of FOT applicability for detecting early smoking associated changes in lungs. This opinion piece will therefore provide a concise analysis on the usefulness of FOT and its clinical utility as a novel diagnostic tool for early detection of small airway changes in smokers. We also explore recent modifications in the conventional FOT that might increase the diagnostic accuracy of the technique for early diagnosis of smoking related airway changes.

## 2. Clinically Relevant FOT Parameters for Detecting Early Changes in Smokers

The use of clinically relevant FOT parameters has varied among the different centres, studies, study populations, and the parameters measured by different commercial instruments. This opinion piece is the first of its kind to describe the clinical utility of different parameters of FOT for early detection of small airway abnormalities in smokers. A number of early studies on FOT failed to differentiate smokers from non-smokers using the Resistance (Rrs) measured by the single frequency or a small range of frequencies [40,41]. Continuing the effort to identify the early abnormalities related to smoking a study using frequency range of 3–9 Hz was the first to show significant differences in Rrs measured at 3 Hz indicating the frequency dependence of resistance [42]. Another study further increased the range of frequencies from 5 to 30 Hz using a random noise technique which showed a significantly higher resonant frequency and a larger frequency dependence of Rrs in smokers [43]. A larger study utilising the frequency range between 4–24 Hz was also unable to differentiate smokers from non-smokers independently however, this study showed the frequency dependence of resistance and reactance parameters [44]. The above mentioned studies have clearly indicated that the sensitivity of FOT for detecting early airflow obstruction is increased with the utilisation of multiple frequencies with differential penetrating abilities of lower frequency (e.g., 5 Hz) reaching down to the peripheral airways, whereas higher frequencies (e.g., 20 Hz) are limited to proximal airways [38]. Resistance (Rrs) at one frequency may not detect subtle abnormalities in smokers but the use of range of frequencies and the frequency dependence of the resistance has been established as a good physiological index for detecting the airway changes in smokers [12,45]. The value of Rrs at low frequency (4–6 Hz) is relatively more useful in differentiating the smoking related airway abnormalities compared to higher frequencies [46]. The reactance (Xrs) also shows most appreciable changes in lower frequencies, mainly in one frequency between 4–6 Hz and resonate frequency. The resonate frequency (Fres), the frequency at which the reactance (X) is zero, and the reactance area (AX5), area enclosed by the negative portion from X (5 Hz) to resonate frequency are also found to be the sensitive markers of respiratory changes in active and passive smokers [47,48]. In earlier studies the smokers and the patients with COPD showed greater Rrs at lower frequencies in accordance with the degree of airway obstruction [14]. However, in more recent study, the mean and the maximal Rrs in lower frequencies was not found to be significantly different between the patients with mild to moderate COPD. Meanwhile, the same parameters were significantly different between the two COPD groups in higher frequencies. These findings may be associated with the upper airway shunt effect, which increases with respiratory impedance and/or oscillation frequency. These findings might be characteristic respective features in mild and moderate COPD, which can be a useful property for diagnosing the early stage of COPD [49].

The range of frequencies used in modern FOT devices also allows independent assessment of both proximal and peripheral airways, where the lower frequency (5 Hz) penetrates down through the proximal airways to the peripheral airways giving the measure of total airway resistance, whereas higher frequencies (20 Hz) relate to proximal airway resistance. Thus, research has interpreted R5–R20 as a marker of peripheral airway resistance and found to be increased in non-symptomatic smokers compared to non-smokers [38,50,51]. Changes in R5 and R5–20 have also been observed in adolescents exposed to maternal smoking and are therefore projected to be a sensitive marker of airway changes in passive smokers [52]. Despite the common finding regarding R5–R20 to reflect the small airway calibre, the recent update of the European Respiratory Society (ERS) recommendation for clinical use of FOT has pointed to the uncertainty in the interpretation of frequency dependence, as there are yet no published data correlating the pathology, and has highlighted the need for further research in this area [53,54]. While this update partially updated the 2003 guidelines on clinical use of FOT for technical standards, it has not included the recommendations on clinical application of FOT in respiratory disease and its potential for differentiating disease from non-disease stating that it was not within the scope of current task force [53].

Studies with modern FOT devices have shown that they are sensitive to detect the dose-dependent changes in airways associated with smoking. Early adverse effects of smoking were more accurately detected by absolute values of respiratory impedance (Z) at 4 Hz followed by resistive impedance at 0 Hz (R0) and respiratory system dynamic compliance (Crs, dyn) [55]. Similarly, another study reported the average resistance between 4 to 16 Hz (Rm) to be the most accurate frequency to detect smoking induced changes [36]. Study in groups of smokers with different smoking history in pack years, the diagnostic accuracy of R0 and mean resistance (Rm) were considered to be adequate for clinical use with sensitivity and specificity to detect changes in smokers with <20 pack years of smoking history [19].

These studies indicate that FOT parameters could be a sensitive diagnostic tool to detect early respiratory changes in smokers. The evidence published to date indicates that multiple frequencies and the frequency dependence of resistance and reactance provide the most valuable information regarding smoking induced changes. However, the optimal frequency combination and clinically most relevant indicator are yet to be determined with further studies with larger sample size.

**Table 1:** Summary table of the studies using Forced Oscillation technique (FOT) to detect early respiratory changes in smokers.

Study/Year	Study Design/Frequency Used/Methods	Summary of Finding
Borrill, Z. L. et al., 2008 [47]	Compared inflammatory markers, spirometry, plethysmography, IOS with multiple frequency and in 18 smokers and 10 non-smokers	Resonate Frequency was significantly higher in smokers (compared to non-smokers while other parameters of IOS were not significant. IOS showed the detrimental effects of smoking while FEV1 was normal
Faria, A. C. et al., 2010 [19]	FOT measurements using AOS (4–32 Hz in170 subjects divided into five groups according to the number of pack-years smoked as <20, 20–39, 40–59, and >60 pack-years and a control group	R0, RM and CRs, dyn values are more useful than spirometry in detecting early changes in smokers. R0 and Rm obtained AUC values considered adequate for clinical to detected change in <20 pack years group
Crim, C. et al., 2011 [30]	Measured lung impedance with IOS (multi frequency) in healthy non-smokers (*n* = 233), healthy former smokers (*n* = 322) and patients with COPD (*n* = 2054) and compared it with spirometry and CT	No differences in IOS between smokers and non-smokers except for R20. IOS was not significantly correlated with smoking pack years. Weak association between IOS and MMEFs was seen. IOS appeared to be more variable than spirometry over the period of 3 months
Shinke, H. et al., 2013 [38]	Intermittent hunning impulse of 4–36 Hz used for comparison of the impedance components in non-smokers, smokers, and COPD patients during inspiratory and expiratory phases	Difference between the maximum and minimum values of R5 and X5 i.e R5 Sub and X5 Sub were significantly different in smokers from non-smokers
Silva, K. K. et al., 2015 [37]	Twenty healthy individuals, 20 smokers and 74 patients with stable COPD were evaluated for the mean respiratory impedance (Zm) and the respiratory cycle dependence in the impedance. Low pressure sinusoidal signal filtered at 5 Hz with analog filter was used	Non-significant changes along the respiratory cycle in healthy subjects and smokers, but significantly higher expiratory impedance values than the inspiratory values inCOPD patients
Berger, K. I. et al., 2016 [31]	FOT with multiple frequency and inflammatory markers were measured in bronchioalveolar lavage from 7 controls and 16 smokers	Smokers had elevated R5 with abnormal R5–20 and X5. Abnormal FOT was associated with two-fold higher lymphocyte and neutrophil counts and with higher interleukin (IL)-8, eotaxin and fractalkine levels
Contoli, M., 2016 [50]	Fifty asthmatic patients (25 current smokers and 25 non-smokers) performed single breath nitrogen wash out and IOS with multiple frequency	R5–R20 but not R5 was significantly higher in smokers compared to non-smoking asthmatic patients. R5–R20 comparable with dN2 in detecting airway changes in smoking asthmatic group
Schivinski, C. I. S. et al., 2017 [48]	Spirometry and FOT using IOS (5–20 Hz) was performed in 6–14 years children divided as passive and non-passive smoker, (*n* = 78)	The passive smoker group had higher mean absolute values for reactance area (AX5) and significantly higher percentage of predicted values for R20, Fres, X5 and AX. IOS was able to identify the early changes in lung function in adolescent and children due to passive smoking
Jetmalani, K. et al., 2018 [56]	Eighty smokers with normal spirometry completed a symptom questionnaire and went for MBNW and IOS using 5–35 Hz pulse of pressure waves	Forty-one (51%) subjects had at least one abnormal IOS parameter, predominantly in resistance. Sixty-one (76%) subjects had an abnormality in either MBNW or IOS. Abnormalities in MBNW and IOS parameters were unrelated to each other
Ribeiro, C. O. et al., 2018 [36]	40 healthy controls, 40 smokers (20.3 ± 9.3 pack-years) and 40 patients with mild COPD performed FOT (4–32 Hz). The contributions of the integer-order (InOr) and fractional-order (FrOr) models was evaluated	FOT parameters and InOr modelling may adequately identify early changes (–AUC > 0.8). The use of FrOr modelling significantly improved the process, allowing the early diagnosis of smokers and patients with mild COPD with high accuracy (AUC > 0.9)
Thacher, J. D. et al., 2018 [52]	Investigated the influence of maternal smoking during pregnancy, second-hand smoke exposure and adolescent smoking on lung function. Participants performed spirometry and IOS (*n* = 2295)	Significant increases in R5, R5–20 and AX0 in adolescent exposed to maternal smoking during pregnancy. Effect of a smoking in adolescent was also shown by IOS parameters
Soares, M. et al., 2019 [57]	Compared two commercially available FOT devices: Impulse Oscillometry (IOS) and TremoFlo FOT (AOS) in (a) healthy controls (*n* = 14), asymptomatic smokers (*n* = 17) and individuals with asthma (*n* = 73) and (b) a 3D printed CT-derived airway tree model	Both R5 and R20 of IOS were able to differentiate smokers from healthy controls but only R19 of AOS was able to do so. IOS consistently measured higher resistance values compared to AOS at both 5 Hz and 20 Hz in all patient group. The printed airway resistance and standardized volume reactance confirmed the observations seen in patients

## 3. Methodological Variation in FOT for Detecting Early Changes in Smokers

The FOT, introduced by DuBois et al. in 1956, is a method for non-invasively assessing lung mechanics by examining the relationship between pressure and flow with forced oscillations delivered to the respiratory system by a loudspeaker or piston [54,58]. Most research groups prior to mid-1990s designed and developed their own instrument [46]. More recently, the number of commercially available and self-made machines has increased, which led to an effort to compare and standardize the measurements between the different devices, however such comparison studies reported remarkable differences between different devices [59,60,61]. These variations in the results may be due to the differences in the methods of forcing oscillation signals, in the device hardware, in the load imposed by the breathing circuit or from the data processing systems used [59,60,61,62].

Several studies have evaluated and established the utility of FOT, using the techniques of Impulse oscillation system (IOS) and Airway oscillation system (AOS) in adults and children. However, there remain discrepancies in FOT values measured by IOS and AOS system in healthy controls [39,61]. There is paucity of studies investigating the ability of FOT to detect the early respiratory changes in asymptomatic smokers and further between-device comparisons in this population is very rare. To our knowledge, the only study that compared the IOS and AOS in healthy controls, asymptomatic smokers and asthma patients demonstrated that IOS consistently measured higher resistance values compared to AOS at both 5 Hz and 20 Hz. These observations were consistent across all groups and in the pooled study population. Both R5, and R20 of IOS were able to differentiate smokers from healthy controls but only R19 of AOS was able to do so and all other measures of both IOS and AOS were not significantly different in two groups. [57]. A potential explanation for the differences across the devices may be the difference in signals used and the data processing algorithm utilized for data processing. In particular, the IOS device employs a harmonic impulse train signal in contrast to the single or composite sinusoidal signal used by the AOS. The signal/noise ratio is more related to the fact that the amplitude signals are more concentrated at fundamental frequency (5 Hz) and the potential harmonic interference from other frequencies at multiples of 5 Hz specific to the IOS signal may also contribute to the differences observed [39,61,63]. Further the respiratory system exhibits non-linearities such as turbulence, volume dependence of respiratory tissue properties, and expiratory flow limitations which may be more evident in smokers or in other obstructive conditions [22,64]. The estimation of impedance in the presence of non-linear conditions is influenced by entire spectral characteristics of forcing signal and waveform which may be another reason for the difference [62]. The other important methodological difference in the above study is the IOS system allowing the measurement of 5 impedance spectra per second that may capture the within breadth variability, where the expiration is believed to be more affected by non-linear phenomenon compared to inspiration [61]. This feature was not available in default setting of the AOS device used in the study (TremoFloC-100), but it has been upgraded now to provide intra breath variability parameters in its customized products which is believed to give somehow similar details of within breath variability as that of an IOS system [65]. The better agreement between impedance value measured by different waveforms but with same algorithm suggests the potential contribution of data processing method for the differences of values measured by different devices [62].

With increasing availability of commercial devices with different design of testing, hardware, oscillation signal properties and post processing properties, further in-between device standardization will be required to choose the suitability among the available devices for deployment in clinical settings. The discrepancies in the results from between device comparisons highlight the need for further validation procedures that also considers the factors including oscillation signal, device hardware, load due to breathing circuit, data processing system and the non-linearities in the respiratory system. The validation procedure could be benefited with the use of a reference test load with known resistance and designed to mimic the breathing pattern in a controlled manner. Defining the standards for data processing and comparison between the devices producing different signals but with similar hardware and data processing system is the other way to make the comparison among the different signal processors. The other approach for validation and quality control in the absence of the above-mentioned designs is to use a large cohort of healthy human controls as a reference.

## 4. Modified Approaches in Data Analysis and Interpretation to Increase the Sensitivity

Over the past decades, a consensus has developed that FOT had a clear clinical utility and can be related predictably to physiological changes in respiratory systems. FOT offers useful information of early airway changes in smokers and other occupational smoke exposure. Despite this consensus, isolated studies have raised the concerns about the sensitivity, specificity and clinical acceptability of the conventional FOT measures [19,38,47,50,56,57]. In mid 1980s researchers investigated the variation in total respiratory system resistance between air breathing and a mixture of helium-oxygen breathing. This was one of the earliest methodological modifications to conventional FOT to increase the sensitivity of FOT to detect early airway abnormalities caused by smoking or occupational exposure. The degree of frequency dependence of resistance and the change in frequency dependence between air and He-O_2_ breathing were the only parameters more sensitive than spirometry to differentiate the smokers from healthy controls [45]. However, no further studies to our knowledge reported the use of air density dependence in FOT and its additional advantage for detection of early airway changes in smokers.

Earlier studies used fast Fourier transformation, cross-correlation or least square technique for signal processing which allows the within breath FOT (WbFOT) measurement with limited time resolution, typically of 0.2 s or 0.25 s. To improve the differential study between the phases of respiratory cycle and derive precise measurements at specific time of interest, researchers have customized the routine FOT with analog signal processing circuits which allow continuous real time calculation of WbFOT [66]. Most commercial FOT devices now implement digital signal processing unit, which can measure continuous, real-time impedance including within breath measurements [67]. A study by da Silva et al. [37] investigated the influence of airway obstruction on within breath FOT in COPD patients and smokers. This study measured mean respiratory impedance (Zm) as well as the impedance values for the inspiration (Zi) and expiration cycles (Ze) at the beginning of inspiration (Zbi) and expiration (Zbe) and calculated the peak-to-peak impedance (Zpp = Zbe-Zbi) and the respiratory cycle dependence (DZrs = Ze-Zi). The study showed non-significant changes along the respiratory cycle in healthy subjects and in smokers, but they have reported significantly higher expiratory impedance values than the inspiratory values in patients with mild, moderate and severe obstruction. These results raised questions on the ability of WbFOT to detect early respiratory changes in smokers [37]. However, the other study utilizing three-dimensional visualization of the respiratory resistance and reactance in smokers along a time axis reported the frequency dependence of resistance during the expiratory phase as an important diagnostic tool for detection of smoking related changes. The differences between maximum and minimum R5 and X5, inspiratory and expiratory difference in R5 (R5sub) and X5 (X5 Sub) were sensitive markers for respiratory changes in smokers [38].

Machine learning algorithms developed from the original FOT values obtained from smokers have shown to have the potential to detect early respiratory changes in smokers with approximately 85% sensitivity and specificity, this algorithm can be useful as clinical decision support system to diagnose early respiratory abnormalities in smokers. [20]. Fractional-order (FrOr) models is the other proposed modification in traditional FOT to provide greater insight of dynamic behavior and peripheral changes in respiratory system [68]. The FrOr model is physiologically interpreted as a frequency-dependent fractional inertia (FrL) which takes into account a constant phase impedance which is a component relative to peripheral airways and frequency dependent fractional compliance (FrC) [69]. This model improved the ability of traditional FOT for early diagnosis of smoking related changes and mild COPD. High diagnostic accuracy, (Area Under Curve (AUC) 0.9) was obtained in smokers with the best FrOr parameter, while traditional FOT achieved only adequate diagnostic accuracy (AUC 0.8) [36].

The use of different gases for breathing, modified data analysis and interpretation in FOT using within breath differential impedance analysis and the mathematical modelling has been trialled to increase the diagnostic accuracy and assist in clinical decision making for early detection of smoking related airway changes. Single studies on each of these modifications showed improved diagnostic accuracy however, non-uniformity in sample size and under reporting of the inclusion criteria for smokers in those studies makes it difficult to compare the value added by these modifications. The choice of different data analysis system and mathematical modelling on FOT for future use should be based on the value added by those systems in standard FOT in terms of comfort to the patients, ease of interpretation and feasibility in the clinical setting. Further studies on each of the above-mentioned systems with larger sample size and standard inclusion criteria for smokers and healthy controls will allow the evaluation and selection of the best modification models which may greatly increase the ability to detect early respiratory changes in smokers.

## 5. Correlation of FOT and Biomarkers in Smokers

Multiple physiological tests have been used to identify small airway abnormalities in smokers; however, the relationship between these abnormalities and inflammation due to smoking remains unclear [9,70,71]. Smokers who did not meet the GOLD criteria for COPD but with abnormal FOT results had more than two-fold higher median neutrophil and lymphocyte cell counts and significantly higher IL-8, eotaxin and fractalkine in bronchioalveolar lavage (BAL) samples compared to similar smokers with normal FOT. FOT markers of distal airway dysfunction (R5–20 and/or X5) also correlated with levels of neutrophils, eotaxin, IL-8, fractalkine, IL-12, p70, macrophage inflammatory protein-1α and growth-regulated oncogene [31].

Similarly, abnormal FOT in smokers was also associated with increased level of 8-isoprostane in exhaled breath condensate (EBC) and sputum neutrophilia. Reduced EBC pH has also been reported in smokers with abnormal FOT and it is correlated with sputum IL-8 [47]. This suggests the link between airway acidity and bacterial colonisation, neutrophilic inflammation and oxidative stress in asymptomatic smokers which was previously reported in COPD patients [72,73,74]. Cigarette smoke is capable of causing oxidative stress and cellular toxicity which may alter the cell function leading to acidification [72]. Further work is needed to elucidate the exact mechanism and importance of airway acidification in smokers. The reports on the value of exhaled nitric acid (FeNO) are quite conflicting, with some reporting it to be reduced in smokers compared to non-smokers while other studies reported no difference in FeNO between smokers with abnormal FOT and non-smokers, and no reduction in the bronchial wall concentration of nitric oxide either [47,74,75,76]. Reports on FeNO are highly variable, being decreased, unchanged or increased even in smokers with COPD indicating it to be a less reliable maker of airway dysfunction in asymptomatic smokers [74,75,76,77]. The possible explanation for this variable level of FeNO is its regulation which is balanced between inhibition of nitric oxide synthase activity by smoking and upregulation of this enzyme activity by inflammation and the amount of NO inhalation during smoking [77].

The analysis of inflammatory, oxidative stress markers in bronchioalveolar lavage or in induced sputum and exhaled breadth condensate along with non-invasive FOT could possibly explain the link between oxidative stress, inflammation, and early airway dysfunction in smokers. Further their correlation could predict disease progression and might help to identify the relevant COPD phenotype [30,78,79].

## 6. Conclusions

FOT could be a versatile diagnostic tool to detect early respiratory changes in smokers while they are still at a potentially reversible stage. Several studies are being conducted in different sample populations with different oscillation techniques and modifications to find the optimal settings for the clinical application of FOT. Currently the major limitation for the clinical use of FOT is the interpretation which requires experience and training. The recent advancement of the developing machine learning algorithm is expected to assist in the interpretation and clinical decision making. The identification of the most sensitive and adequate parameter of FOT to detect early respiratory changes in smokers, together with its documentation in the official guidelines, is the next key step for its application in clinical settings. Considering its less invasive nature, comfort to old and disabled patients, feasibility in clinical setting and cost effectiveness, it could be the first-choice diagnostic technique for detection of early respiratory changes in smokers. Further longitudinal studies with FOT and other diagnostic tools including spirometry and blood markers (like matrix metalloproteinase MMPS and its inhibitors, and club cell protein 16 (CC16)) comparing smokers and non-smokers for the development of COPD and other respiratory pathologies can be useful for understanding disease progression. The finding of FOT could further be supported and correlated with inflammatory markers from bronchioalveolar lavage, exhaled breath condensate or sputum for better understanding of airway pathogenesis in smokers.
